# Patient participation in discharge planning conference

**DOI:** 10.5334/ijic.1543

**Published:** 2014-11-06

**Authors:** Angela Bångsbo, Anna Dunér, Eva Lidén

**Affiliations:** Institute of Neuroscience and Physiology, Department of Clinical Neuroscience and Rehabilitation, The Sahlgrenska Academy, University of Gothenburg, Gothenburg, Sweden; FoU Sjuhärad Välfärd, University of Borås, Borås, Sweden; Department of Social Work, University of Gothenburg, Gothenburg, Sweden; Institute of Health and Care Sciences, The Sahlgrenska Academy, University of Gothenburg, Gothenburg, Sweden

**Keywords:** patient participation, hospital discharge, frail older persons

## Abstract

**Introduction:**

There is a need for individualized discharge planning to support frail older persons at hospital discharge. In this context, active participation on their behalf cannot be taken for granted. The aim of this study was to elucidate patient participation in discharge planning conferences, with a focus on frail older persons, supported by the theory of positioning described by Harré & van Langenhove.

**Methods:**

The study was designed as a case study based on audio-recordings of multidisciplinary discharge planning conferences and interviews with health professionals elucidating their opinions on preconditions for patient participation in discharge planning. The analysis has been performed using qualitative content analysis and discourse analysis. Data collection took place during 2008–2009 and included 40 health professionals and 13 frail older persons in hospital or municipal settings.

**Results:**

Findings revealed four different positions of participation, characterized by the older person's level of activity during the conference and his/her appearance as being reduced (patient) or whole (person). The positions varied dynamically from being an active person, passive person, active patient, or passive patient and the health professionals, next-of-kin, and the older persons themselves contributed to the positioning.

**Conclusions:**

The findings showed how the institutional setting served as a purposeful structure or a confinement to patient participation.

## Introduction

When being discharged from hospital, a participative patient has a positive impact on establishing safe and secure care, which increases patient satisfaction, reduces the risk of readmission and strengthens the patient role within the organisation [1–4]. These needs often require integrated, coordinated care where collaboration between care providers is necessary [5]. This study aims to elucidate patient participation in discharge planning conferences with a focus on frail older persons and the opinions of health professionals regarding preconditions for patient participation in these conferences. Frail older persons often have multiple diseases affecting biological, psychological and social functions with complex and comprehensive care needs [5, 6]. Discharge planning aims to ensure care continuity and identify possible social care needs, and has been defined as holistic rather than only focusing on medical needs, as well as being a process in several stages [7]. It involves the patient, carers and family. Communication is important and when it is successful, it results in appropriate decisions being made in relation to available care resources [7]. In this study carried out in a Swedish context, the concept ‘discharge planning conferences’ refers to a multi-professional meeting aiming to coordinate care in hospital, a community dwelling or municipal health and social care settings. The participants in these conferences vary but primarily involve the patient, next of kin and health professionals from the hospital, municipal health or social care facility, or in special cases from the primary health care centre. The manner in which they are carried out depends on local circumstances.

In discharge planning conferences, patient participation is dependent on empowerment and knowledge of the conference, the patient having a communicative ability to influence decision-making according to his/her individual needs and an ability to show interest in the situation [8]. However, there is current evidence indicating that discharge planning from hospitals can be improved. Shorter care periods and the working situations of health professionals often result in planning *for* the patient instead of *with* the patient [8, 9].

The concept ‘participation’ has many definitions where some are confining [9] or simply comprise participation in decision-making such as in MeSH [11]. In this study, we have found support in the definition from international classification of functioning, disability and health (ICF) [12], where participation is broadly defined in a health-related context, from a holistic view, as actively taking part in a life situation. Findings explore health professionals’ views on patient participation in discharge planning conferences based on clearness of aims and outcomes, the importance of inclusive practices, person-centredness and mutual relationships between participants [13]. The views of frail older persons on participation in medical decision-making mainly support Donnelly's [13] study, although some felt uncertain due to their role as a patient in the discharge planning conference, doctors’ attitudes, the health care system and logistical factors [14]. A review of research within the area shows that many studies have examined the effects, preconditions and hindrances of participation [15–17]. However, there is a lack of research on *how* patient participation in these conferences is accomplished. Well-functioning discharge planning conferences where patients participate actively are dependent on communicative skills that need to be made visible [18–20]. Therefore, variations in communicative positions taken by patients and health professionals in discharge planning conferences will be elucidated.

Positioning theory constitutes an approach to understanding institutional talk between health professionals and laymen, by describing what happens between individuals, how they act in relation to others and what communicative positions appear in institutional conversations. According to positioning theory, discharge planning conferences could be seen as situations where the participants interactionally position themselves and each other resulting in a power asymmetry that might invite or confine patient participation [21]. Person-centredness presupposes an understanding of group processes that improves older persons’ possibilities to participate in institutional conversations [22]. Research about institutional conversations shows that patients are being ‘processed’ within organisations, in order to raise reactions from other social units and in order to rationalise health professionals’ work within the institution [23]. Discharge planning conferences are thus institutional arenas where health professionals in hospitals transfer care responsibilities to primary and municipal health and social care facilities. In health care institutions, frail older persons are classified as ‘patients’. This classification, however, increases the risk of reducing persons as a whole and decreases their possibility to actively participate in the planning of health care [23, 24].

Because of the problematic issue of the implications of people categorisation, the informants in this study are titled ‘frail older persons’ and ‘health professionals’. All informants are referred to as ‘her’ as there were more women represented in the data than men. The informants titled ‘health professionals’ represent primary, secondary and municipal health and social care workers.

## Aim

The aim of the study was to elucidate the variation of frail older persons’ positioning in discharge planning conferences. Moreover, the study aimed at elucidating health professionals’ opinions on the preconditions for frail older persons’ participation in these conferences.

The specific research questions are:

How do frail older persons position themselves at discharge planning conferences?

How do health professionals contribute to this positioning?

What are the opinions of health professionals on facilitating or obstructing conditions related to patient participation in discharge planning conferences?

## Methods

This study describes discharge planning conferences from the perspective of the health professionals and uses the *case study* methodology by Stake [25]. The study was designed as a multiple *case study* to gain knowledge about the phenomenon ‘patient participation’ by analysing the case ‘discharge planning conference’. In this study, discharge planning conferences are seen as communicative events representing a kind of institutional meeting [26]. They are held formally ‘front stage’ where health professionals, the frail older person and his/her next of kin are present. However, talks and activities are also running inter-professionally ‘back stage’ before and after the conference, and what is discussed and decided then is often not discussed during the actual discharge planning conference [27, 28]. This is in line with Stake's [25] definition of a case as a system consisting of different parts, where some are within and some outside the case [25]. A *case study* represents both the research process around the case and the outcome.

### Data collection

The multiple sources in this study were represented by audio recordings as primary data and interviews reflecting the views of health professionals as secondary data (see Table 1). Data triangulation was performed in several steps by two of the authors (AB and EL). Data was collected from 2008 to 2009 and consisted of interviews with health professionals and audio recordings of discharge planning conferences. Health professionals in this study refer to those working in secondary and municipal health and social care. Based on their experiences, they reflected upon the actions taken by frail older persons and the health professionals’ actions connected to discharge planning. The audio-recorded discharge planning conferences took place in the patients’ own homes, in hospitals or municipal settings and were run by health professionals both with and without discharge planning specialised teams during the period of August 2008 to February 2009. The interviews with health professionals were conducted during the same period, when possible at their workplaces but otherwise at the researchers’ office.

### Selection of informants

The discharge planning conferences (DS1) were selected using purposive sampling by nurses on the hospital wards and social workers in the community. Frail older persons aged 77 years or older with at least two health-related problems, with the ability to communicate and without cognitive or hearing deficits, were included. They had a need for further nursing, rehabilitation and/or care in the home. The data consisted of 13 conferences including 4 men and 9 women, aged 77–89 years. The frailty or vulnerabilities of older persons may have caused recruitment problems. Three discharge planning conferences had incomplete recordings so a total of 10 conferences were included in the sample consisting of 7 women and 3 men, 2–7 health professionals, 0–3 next of kin and ranging in length from 19 to 57 minutes.

The health professionals participating in the interviews (DS2) were selected so as to obtain geographical and institutional distribution. They were recruited using purposive sampling with help from the managers of the care organisations. The health professionals included 40 participants; 20 nurses, 4 physiotherapists (PTs), 6 occupational therapists (OTs) from municipal health and social care and secondary care, and 10 social workers.

### Data collection informants

#### DS1 audio recordings

Nurses and social workers first asked suitable patients to participate in the study and gave potential participants an information letter before their discharge planning conference (DS1). In order to get informed consent, the frail older persons deemed suitable for the study were visited by one of the researchers prior to the data collection, in order to get the opportunity to ask questions about the study. Next of kin *and/or* health professionals that could be involved in the study were informed about the study by telephone. In one case, the next of kin refused participation. Specialised teams ran 6 of the 10 discharge planning conferences. Two departments refused participation due to too few discharge planning conferences, department reorganisation or heavy workloads. There was variation regarding how the conferences were carried out. A short meeting preceded some of them, where a nurse at the hospital gave information from the care period without the frail older person being present. After that, the discharge planning conference proceeded without the nurse's presence. Other discharge planning conferences were preceded by a telephone conference with the frail older person participating. The researchers (EL and AB) collected all data.

#### DS2 interviews with health professionals

The health professionals were contacted by phone *and/or* e-mail and then received written information on the study before they decided whether to participate or not. In addition, they chose where the interview was going to take place; the majority was held at their workplaces and in two cases at the researchers’ office. To support the interviews, an interview guide with questions regarding the health professionals’ views on patient participation at discharge planning conferences was used. Five persons, the researchers (EL and AB), one research assistant and two master's students, performed the interviews with the health professionals using a guide that was based on the results of previous research on discharge planning, developed by the researchers. The profession of the interviewer was never the same as the informant, to avoid any overly collegial conversation (DS2). The researchers transcribed all data verbatim, DS1 and DS2.

### Data analysis

The data analysis had two main focuses. The first focused on how the variation of frail older persons positioning was established in interaction between health professionals, frail older persons and next of kin during discharge planning conferences. The second focused on the health professionals’ opinions on the preconditions for the participation of frail older persons in discharge planning conferences.

#### Analysis of DS1

**Figure 1.**
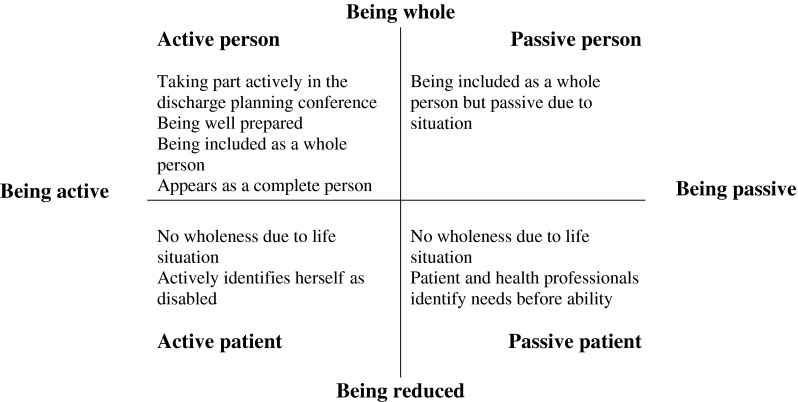
Overview of frail older persons’ positioning in discharge planning conferences.

The analysis of DS1 was inspired by Roberts and Sarangis’ [29] model of theme-oriented discourse analysis. The audio-recorded discharge planning conferences were processed in four steps. In step one, one of the authors (AB) transcribed the audio recordings, and all authors read the transcriptions to get an overall idea of its content and they shared their reflections. After this, an analysis focused on *how* the participants communicated in the setting of the discharge planning conferences (step two). The thematic concepts ‘interactive frames and footing’, ‘contextualisation cues and inferences’, ‘face and face work’, ‘social identity’ and ‘rhetorical devices’ were thus used as analytical tools [29]. In the third step, the analysis aimed at discovering if and how power asymmetries in interaction were apparent [21]. Two dimensions of the data were thus coded based on the older person's activity (codes = active/passive) and representations of data which highlighted the older person from a holistic perspective or based solely on limited aspects (codes = subject/object). Finally (step four), data were categorised and synthesised, and resulted in a model (Figure 1) aiming to illustrate the variation and dynamics of the positioning of the frail older persons.

#### Analysis of DS2

The health professional interviews were analysed in four steps using qualitative content analysis with a deductive approach, on the basis of the results from the discourse analysis with an aim to elucidate health professionals’ opinions on the preconditions for frail older persons’ participation in discharge planning conferences. Initially, data were transcribed verbatim and then read to get an overview of the content. In the third step, it was elaborated on the basis of the results from the analysis of DS1 data to identify meaning units related to issues of activity/passivity and opportunities of being presented as a person or patient in the context of the discharge planning conference [30]. Data that could not be coded were left out, identified and analysed later in the fourth step to decide if they represented a new code or subcategory, supported by a directed approach to content analysis [30]. The results are presented in two parts: how frail older persons’ positions were constructed at the discharge planning conferences and how the impact was understood by the health professionals.

## Ethical considerations

Ethical approval for the recordings of the discharge planning conferences was obtained from the Regional Ethical Board (Dnr: 816–08). The vulnerability of frail older people has been taken into consideration during the research process and caution has been taken not to underestimate their capability [31]. There was no need for ethical approval according to Swedish law concerning the interviews. The participants gave informed consent. The data were handled confidentially and only the researchers had access to them.

## Results

The results of this study showed how the participation of frail older persons in discharge planning conferences was communicatively constructed as their care needs and suitable activities were negotiated. This was a process that simultaneously involved positioning the frail older persons. How the positioning occurred depended on how the frail older persons acted and the approach of the health professionals. According to the health professionals, the positioning is also related to contextual factors that characterised the institutions involved in the discharge planning process.

### Positioning of frail older persons during the discharge planning conferences: Being positioned or taking position?

Data showed that frail older persons took one of four different positions during the discharge planning conferences (Figure 1). The positions were established either by the frail older person, the health professionals (HPs) or next of kin, and varied dynamically between the different categories during the discharge planning conference from being an (1) active person, (2) passive person, (3) active patient or (4) passive patient (see Figure 1). How the positioning was established is further described below.

#### Being positioned as or taking the position of an active person

The frail older person in this position appeared to be engaged in the situation, expressed her wishes to the others, and emphasised her narrative to show who she was or had been to portray herself as a whole person. In this position, the frail older person ‘made’ herself active, was well prepared or had some prior understanding or knowledge of the topic and even initiated and led the discussion. She was reflective and eager to know what kind of help she could be offered after discharge, as for example one older person asked:

The bed that I have, do you have something I can put behind my back so I can sit up in bed?

This quote indicates that she had prepared herself for the discharge planning conference, and that she knew there were technical aids available such as back supports for bed use and that she had a need for one due to earlier habits. In order to help frail older persons become involved in the conversation at the discharge planning conference, the health professionals used the strategy of giving some brief information initially and then inviting her into the conversation by saying, for example:

Have you thought of anything?

The health professionals expected the frail older person to contribute to goal formulations and estimate functional ability after discharge. The health professionals and the next of kin contributed to this positioning when acting in a way that stimulated activity, such as by asking open-ended questions that promoted activity and reflection. Being in this position seemed to be a strength when the next of kin or the health professionals had different opinions to the frail older person.

#### Being positioned as or taking the position of a passive person

In this position, the frail older person made efforts to present herself as living in a personal context, involving her own life stories, personal preferences and being in need of autonomy and integrity. She was eager to emphasise individual needs and wishes and to demonstrate the ability when saying, for example:

I live in a house. On my own.

However, her position could be marked as passive due to weak health status or passivity in interaction at the discharge planning conference, for example:

I will have to ask my next of kin, my daughter, if she's at home, if they can cook me some meals. It's very hard to know one's capability before one gets home.

This person had problems estimating her capability after discharge but put trust in her next of kin to support her. This position also occurred when the frail older person delegated decisions to other participants such as her next of kin or the health professionals, or in cases where they had difficulties estimating the situation post-discharge. As an example, the following short quote illustrates how a frail older person reflected on the past without any proposals for the future:

It will not be like before.

Face-saving strategies were used in order to protect integrity, show themselves as unique individuals or keep up an image of being an active citizen in society. In situations where the discourse threatened personal integrity, such as when accepting help to take a shower, joking appeared as a strategy to bring a sense of being a person that maintains integrity:

Scrubbing my back is good too!

It was also seen that the health professionals and next of kin supported the frail older person as a ‘passive person’, in a dialogue characterised by carefully keeping her as the focus of the discharge planning conference when health professionals said:

I think that initially, as you have been quite ill, it can be a good idea to have someone to look after you.

The older person was in focus but being passive when asked to estimate her capability, and the health professionals would decide before her.

#### Being positioned as or taking the position of an active patient

In some cases, it was obvious that the focus of the discharge planning conference was primarily on the frail older person's functional abilities and disabilities. Her personal life story and her capabilities were not discussed either by the health professionals or the frail older person herself. This kind of positioning is illustrated by the following quote:

I hurt my leg; therefore I cannot use it today’, where the speaker focused only on her disability.

Moreover, data showed that frail older persons were active mainly when describing anamnesis or the treatment course given by the doctor:

It was so-so, they didn't treat it so much, it's supposed to heal on its own. Then it is important to get a good medical treatment not to strong or too much and that I can agree with, and that will be efficient and I think they've done a really good job there.

They would also ask for next of kin to support them with different kinds of tasks such as tidying, but have problems motivating their standpoints and comprehending their forthcoming home situations. Participation was evident in situations where the health professionals adopted an approach that facilitated the patient's possibilities to be active, though participation was obstructed when the ‘patient’ position was supported.

#### Being positioned as or taking the position of a passive patient

Frail older persons who appeared as ‘passive patients’ ignored or were deprived of opportunities to participate, when assessing or planning care needs. Only limited aspects were regarded. Data revealed that frail older persons in these cases contributed with descriptions of their functional ability from an outside perspective where the body was described as an object. Lack of confidence in the future and low interest among patients was significant in this category. This position also occurred in situations when next of kin spoke for the frail older person or dominated the discharge planning conference. Sometimes opinions differed concerning care needs and they talked ‘over her head’, excluding him from the conversation:

He really wants to be able to walk again.

In other cases, next of kin influenced the decision-making by taking on a passivising approach towards the frail older person and sharing integrity-threatening information that she had left out herself, e.g. regarding incontinence:

She needs cellulose wadding too (for incontinence care).

It was notable that this approach towards the older person effectively diminished further participation from the older person. The health professionals also contributed towards the frail older person taking on a passive role by asking, for example:

‘How much help do you need to get dressed?’ assuming that she was lacking ability or by using ‘over the head’ talk.

Communicative strategies excluding the frail older person and interrupting her desires and narrative contributed to the obstruction of her participation and her position became passive.

### The impact of the institutional context on the positioning of persons in discharge planning conferences

This section presents empirical findings from the interviews (DS2) on the health professionals’ views on patient participation in discharge planning conferences. The analysis revealed contextual factors that the informants experienced as promoting patient participation when helping frail older persons maintain an image of ‘being an active person’. However, the informants also identified obstructing factors.

#### Promoting factors to positioning as active persons

From the health professionals’ perspectives, data showed that the discharge planning conferences had a purposeful structure in supporting patients in being active and keeping focus on the frail older person and next of kin's needs while giving the frail older person the prime role in decision-making. In order to support the frail older person and next of kin, the health professionals needed to create a calm, secure and well-structured atmosphere, give a sense of continuity, be appropriately skilled and acknowledge and make clear various professional roles and authorisations. A well-functioning inter-professional collaboration was seen as essential to enable a holistic approach. According to the informants, it was helpful if the frail older persons were knowledgeable about the function of discharge planning conferences and what resources municipal health and social care could offer. They recommended that frail older persons should get help in preparing questions beforehand and to summarise what had been decided at the conference afterwards. They found it essential to keep the conversation at a level that the frail older person could understand:

‘Please interrupt me if I say something you don't agree with’ is an example of what one health professional (nurse) said to invite the frail older person to participate actively and as a means of supporting active person positioning.

Finally, informants emphasised the importance of collaboration between professionals and organisations to solve the problems that could occur after hospital discharge, such as those associated with medication, as a way of establishing a safe and secure situation for the frail older person and her next of kin.

#### Obstructing factors to the frail older persons’ positioning within the institutional context

The informants stated that from their experience the positioning of an active person is obstructed if health professionals and next of kin do not facilitate the individual in becoming active and if she is not properly prepared for discharge. Other pitfalls described involved situations at discharge planning conferences where previous family relations affect the way next of kin act towards the older patient at the discharge planning conference or where health professionals expected too much from next of kin as carers resulting in discussions where the frail older person might feel that she had become a burden.

From an organisational perspective, obstructing aspects to active person positioning were caused by lack of collaboration due to insufficient knowledge among health professionals with regard to other organisations involved in the ‘chain of care’. Examples of failures depending on ignorance that resulted in irritation and insecurity among health professionals from different organisations were described in data as having an indirect (or direct) influence on frail older persons’ positioning in a passivising or reductionist way.

Although the informants were aware of the importance of patient preparation before discharge planning conferences, they were unable to suggest how this could be accomplished and which health professional should be responsible for it. However, preparing frail older persons for their upcoming discharge planning conference is considered important, as discharge planning is becoming a more specialised task involving more specialised health professionals, so it is important that individuals are prepared so as to obtain as much as possible from their discharge planning conference. Therefore, to put preparation routines in place would be beneficial. If there are too many participants in the discharge planning conference, the frail older person is at risk of taking a passive position. Some evidence of this was found in the data where the informants described conferences where the patient asked at the end of the meeting:

But what did we decide?(nurse, DS2).

Some informants claimed that the institutional context of discharge planning conferences can be obstructive to patient participation due to limited possibilities for frail older persons to participate, to influence decisions, or to make active choices:

The outcome of the discharge planning conferences seemed predetermined, giving a sense of false participation. (occupational therapist, DS2)

Data also included contrary factors, such as cases where both health professionals and frail older persons were poorly prepared. Finally, the informants emphasised that one of the most obstructing factors to patient participation is when other participants use ‘over the head’ talk or if next of kin dominate the outcome without consulting the frail older person.

## Discussion

This study found that patient participation in discharge planning conferences is possible under certain conditions. However, obstacles also exist such as when the institutional context becomes confining. Patient participation in discharge planning conferences became possible when health professionals offered individuals support and they made themselves active.

The results show that there was an awareness of how to run discharge planning conferences in a way that helps frail older persons become more active and participative but that this was not always possible due to a lack of knowledge, and organisational or practical conditions. To participate as an ‘active person’ was possible if a supportive and communicative approach was used by the health professionals, and if the frail older person used their own approach and was prepared. Positioning is determined when people interact [32] and can be understood as a way of creating discourses from narratives, make them understandable and show how the participants take different positions during institutional talk [21]. Using the pronouns ‘you’ or ‘we’ can support patients in taking more positive positioning to appear as persons [33]. In the data collected in this study, this was obvious when communicatively skilled health professionals spoke directly to the frail older persons, avoided ‘over the head’ talk and listened to what she had to say. Frail older persons appeared as either active or passive and were then categorised as patients or persons with or without support from the health professionals. In human service organisations, people are processed to fit into what the organisation demands and categorised as patients, something that was evident in this study [22]. This processing was evident during the discharge planning conferences, for example, when a health professional used a passivising approach towards the frail older person assuming she was less capable and in need of help. The categorisation expresses a power advantage from the health professionals in relation to the frail older person where the older person is dependent on the services offered by the organisation. The individual has fewer choices than the organisation that has more patients to consider [22].

Discharge planning conferences are held ‘front stage’ but much of the work is also run inter-professionally ‘back stage’ [27] before and after the discharge planning conference, which was evident in the data collected. The health professional informants expressed a feeling that patient participation is hard to achieve, as the outcome of discharge planning conferences is sometimes predetermined due to the health professionals collaboration ‘back stage’. This contradicts person-centred care where the patient should be participating in decision-making [34]. To establish person-centred care, it is essential for health professionals to support shared decision-making and listen to frail elderly individuals’ own narratives [35]. The informants emphasised that discharge planning is becoming an even more specialised task where patient participation cannot be taken for granted, and furthermore, in person-centred care where the patient should have an active part in care and decision-making, be able to take responsibility for herself, and take an active approach. To obtain this, information and knowledge are essential and the patient must be well prepared [36, 37].

There is a greater opportunity for frail older persons to get involved in the discussions when fewer participants at the discharge planning conference with less parallel talk [38]. However, there are also studies indicating that healthcare organisations obstruct person-centred care and health professional education has limited effects without organisational space [39]. Instead, care leadership is important in establishing good care quality, and democratic and inclusive approaches are important to establish person-centred care relations [40].

Studies have shown that health professionals fail to engage patients in discharge planning as they lack explicit organisational guidelines [41, 42]. In this study the informants claimed there was a lack of patient preparation guidelines, questioning who is responsible when discharge planning involves more specialised health personnel. Another possible reason may be that patients’ and health professionals’ opinions on participation differ where patients emphasised participation from a broader definition of collaboration, knowledge, and empowerment, in contrast to only being informed or participating in health-related decision making like in MeSH [11, 43]. Patients expect nurses to optimise participation as it is looked upon as both valuable and important, but they may also have traditional expectations of being informed and accepting the health professionals’ suggestions [44, 45], and in this sense the structure may serve as a confinement. These different explanations may influence the patients’ positioning in the data, depending on expectations [21]. In this study, data has been handled systematically and a certain structure has been identified illuminating possibilities or obstacles for patient participation in discharge planning. It has been validated in different parts by health professionals and health organisation managers through written and oral presentations, and operated by the same project leader during the whole study. A variation of organisations, units, and health professionals in the data is well represented and sufficiently related to the chosen methods.

### Limitations

The frailty of older persons or vulnerabilities such as weakness, fatigue, and cognitive deficits may be a reason for why there were problems recruiting individuals for the study. Even a minor increase in stress could reduce capacity significantly, which might be the case when in hospital care [46]. Although we did not get access to as many discharge planning conferences as intended, our opinion is that the results are not generalisable but the positioning model active person vs. passive patient is transferable to other discharge planning conferences and other target groups. However, the distribution within the positions might vary.

## Conclusion

This study illuminates that patient participation in discharge planning conferences is possible under certain circumstances, but there are obstructing factors within the organisations. Explicit guidelines need to be established in terms of how to inform patients about the need to be prepared for a discharge planning conference and how to limit the number of participants at a discharge planning conference without reducing the decision basis. Inter- and intra-organisational professional collaboration needs to be improved, and structural barriers need to be acknowledged in order to put the patient in focus and encourage participation. Arenas need to be established for health professionals to meet, discuss, and gain possibilities to improve collaborative and communicative skills in discharge planning conferences. Furthermore, health professionals must be given opportunities to develop communicative skills in order to support the active participation of frail older persons.

## Figures and Tables

**Table 1.**
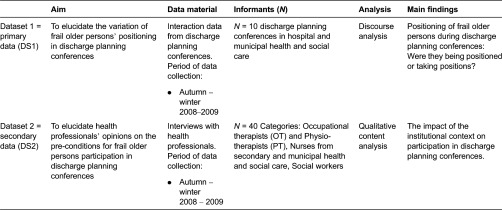
Overview of data sets and analysis.
